# Supplementary material for: Millimeter-scale focal length tuning with MEMS-integrated meta-optics employing high-throughput fabrication
Source: Sci Rep. 2022 Mar 30;12:5385. doi: 10.1038/s41598-022-09277-8 (PMC8967899; doi:10.1038/s41598-022-09277-8)
Supplement: Supplementary file 1 — Supplementary Information. [file 41598_2022_9277_MOESM1_ESM.pdf]

Millimeter-scale focal length tuning with MEMS-integrated meta-optics employing high-throughput fabrication: supplemental document

1. Alvarez meta-optics design dimensions

Table S1 lists the design dimensions for the two complementary Alvarez meta-optics, including the square array layouts and the individual nanoposts used to map the cubic surface profiles. The six linear steps of nanopost duty cycles and their corresponding diameters are selected from the simulated transmitted coefficients (presented in Figure 1 of the main paper) to produce a near-unity amplitude and a phase range from 0 to 2π.

Square Array		
Aperture Size	500 μm × 500 μm	
Lattice Constant	1.3 μm	
Post Height	2 μm	
Individual Elements	Duty Cycle	Diameter (μm)
Nanopost 1	0.3948	513.3
Nanopost 2	0.4316	561.1
Nanopost 3	0.4991	648.8
Nanopost 4	0.5932	771.1
Nanopost 5	0.7404	962.5
Nanopost 6	0.9019	1172

Table S1. Design dimensions of the silicon nitride cylindrical nanoposts for the pair of complementary Alvarez meta-optics.

2. Device images and tuning data for Alvarez lens with actuator Design 2

Here we present the results of an Alvarez metalens fabricated with the same optical elements as the one presented in the main paper but different parameters for the actuator design.

1.1 Fabricated device

Design 2 here has the same Alvarez metasurfaces as Design 1 presented in the main paper. In contrast, the Design 2 electrostatic actuator for the mobile metasurface has a higher spring constant due to its shorter springs of 500 μm and wider finger width of 3 μm. Given the same comb-drive footprint and finger gap, Design 2 has fewer finger pairs involved in actuation. Table 1 of the main paper summarizes the complete comparison between actuator Designs 1 and 2. Compared to Design 1 presented in the main paper, although Design 2 requires a higher voltage to produce a similar focal tuning range, the higher stiffness also leads to a higher natural frequency, giving a more extensive range of actuation frequencies before reaching the instability regime. The differences here show that the users can flexibly modify the actuator designs to boost specific attributes to fulfill different application requirements without changing the device footprint.

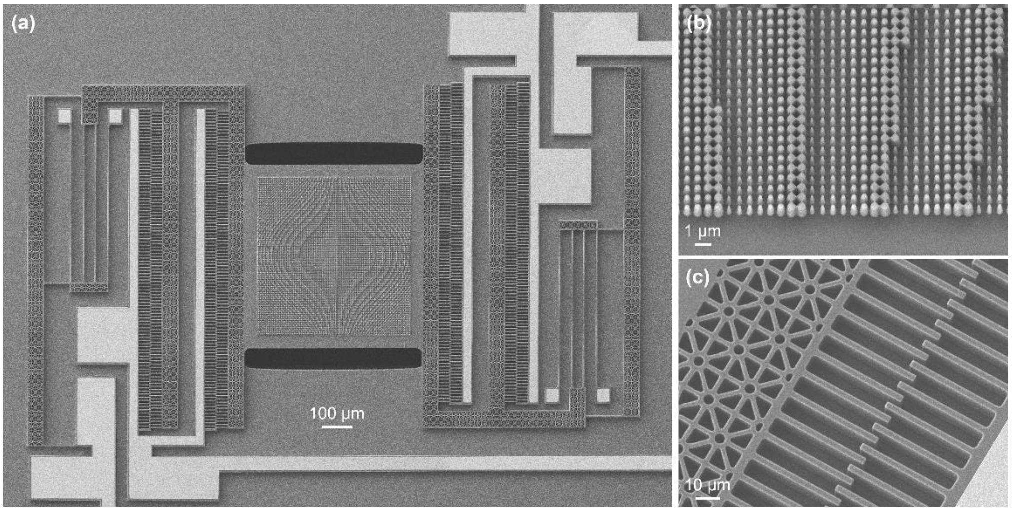

Figure S1. Device fabrication. (a) SEM image of an Alvarez metasurface integrated with a MEMS tuning platform. (c) Close-up view of the silicon nitride nanoposts sitting on the central silicon platform. (d) Comb-drive details showing part of the mobile flexure backbone and interdigitated finger array.

1.2 Electrostatic tuning

Similar to Design 1 in the main paper, the actuated displacement in actuator Design 2 follows the voltage squared curve with negligible hysteresis in Figure S2(b). Figure S2(c) plots the actuation on both the device's left and right sides, and all the data points lie close to the theoretical quadratic fit between displacement and voltage. Calculation from the fit gives a measured lateral spring constant of 2.29 N/m. With the analytical perpendicular stiffness being 45800 N/m, Design 2 is shown to have a stiffness ratio as high as 20000, promising guided uniaxial displacement.

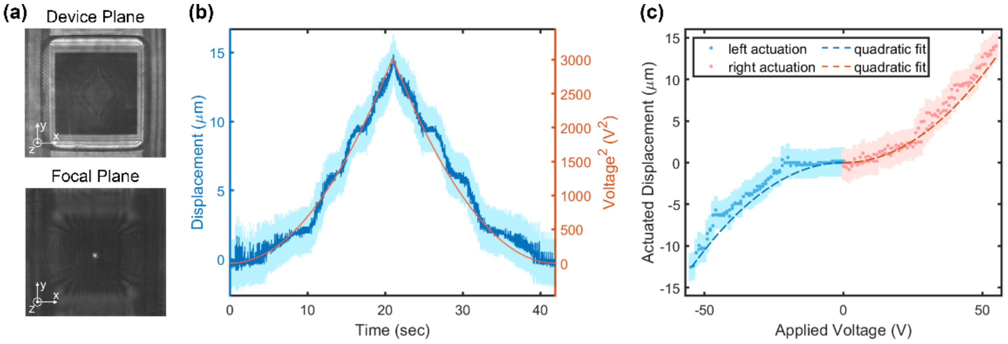

Figure S2. Experimental setup and MEMS performance of the Alvarez lens. (a) Exemplary device and focal planes of the Alvarez metalens actuated at 40 V towards the right. (b) Measured actuated displacement and actuating voltage of the MEMS follow the same trend closely, showing negligible hysteresis. (c) Actuated displacement for both directions follows the theoretical quadratic behavior closely. The light shade bands indicate the associated error bars.

1.3 Alvarez focal tuning

As shown in Figure S3(a), the increasing actuation voltage and actuated displacement shift the focal profile closer to the device plane and concentrate it to a smaller region. Figure S3(b) plots the displacement and resultant focal length as functions of the voltage, while Figure S3(c) plots the focal length and the displacement against each other, showing a close fit to the theoretical Alvarez tuning behavior.

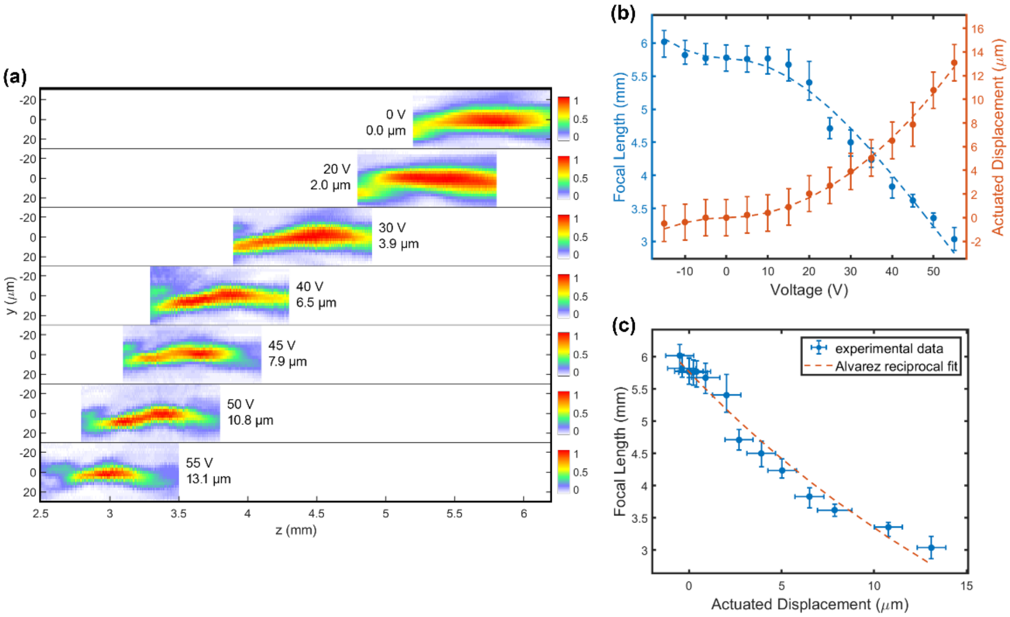

Figure S3. Focal tuning measurements of the MEMS Alvarez metasurface lens. (a) Normalized focal profiles along the optical axis (z). (b) Actuated displacement and the corresponding focal length modulated by actuation voltage. Dashed lines show fitting to theoretical models. (c) Tunable focal length as a function of actuated displacement closely follows the theoretical reciprocal Alvarez tuning behavior.

3. Power consumption calculation

For the MEMS tunable Alvarez meta-optic lens presented in the main paper, we calculate the DC power consumption based on the measured current values and estimate the power consumption per switching based on the measured spring constants and design capacitor dimensions.

2.1 Static operation at DC voltage

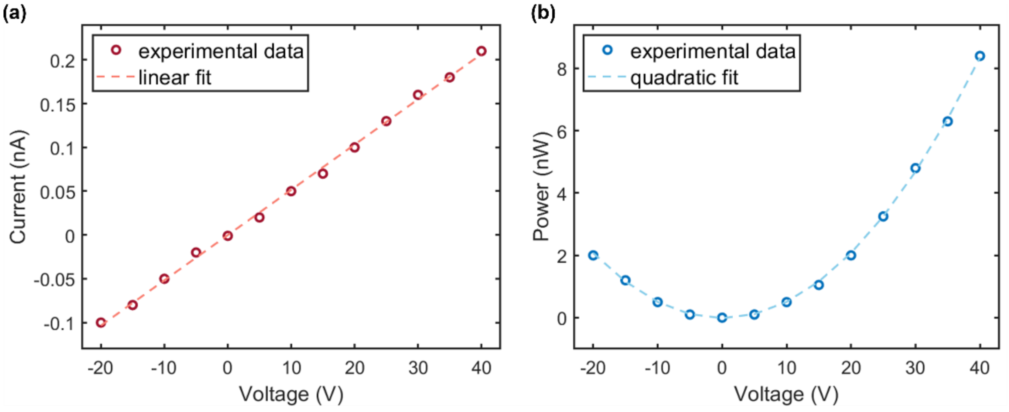

Figure S4. (a) Measured current and (b) calculated power of electrostatic actuator.

While various DC actuation voltages are applied between 0 V and 40 V on the devices with actuator Design 1 (present in the main paper), the resultant currents are recorded as shown in Figure S4(a), which are only several nano-amperes, agreeing with the theory of electrostatic

actuation. The source of DC current is probably the leakage current through the substrate. As described in the main paper, negative voltage values indicate the application to the left comb-drives, while the positive voltage values indicate the application to the right comb-drives. The voltage application on the left side does not continue much further once the focal spot is too stretched and dim to evaluate accurately. The power consumption of the device is calculated as the product of voltage and current

$$P = V \times I = \frac{V^2}{R}. \tag{1}$$

Figure S4 plots the power calculated as the product of measured current and actuation DC voltage. The calculated power closely follows the theoretical quadratic dependence on the applied voltage.

2.2 Energy consumed per switching

Besides the static power calculated in Section 2.1 for a device held at a constant voltage, we expect a small amount of power consumption whenever the voltage switches and the capacitive energy stored in the comb-drive is built up or released.

There are N = 252 finger pairs on each actuation side of the device presented in the main paper, and the comb drive dimensions are summarized in Table 1, where finger height h = 11 µm and finger gap d_{sep} = 2 µm. The initial finger overlap is l_0 = 5 µm. For estimation, we will calculate the largest power consumption at the highest application voltage V = 40 V, which induces a maximum lateral actuation of Δd = 18.1 µm. With the dielectric medium being air, when the actuator is at its maximum displacement, the capacitance in the comb drive is

$$\begin{aligned} C &= 2N\frac{\varepsilon(l_0+\Delta d)h}{d_{sep}} \\ &= 2\times 252\times\frac{(8.854\times 10^{-12}\,F\cdot m^{-1})(5\,\mu m+18.1\,\mu m)(11\,\mu m)}{2\,\mu m} \\ &= 0.567\,pF \end{aligned} \tag{2}$$

and the stored capacitive energy is

$$W = \frac{CV^2}{2} = \frac{(0.567\,pF)(40\,V)^2}{2} = 45.4\,nJ. \tag{3}$$

Assuming the actuator is operating right below the natural resonance f_0 = 1300 Hz (see Section 4.1 in the main paper) before instability sets in, the maximum power consumption can be estimated from the energy required to charge the capacitor per period

$$P = \frac{W}{period} = \frac{45.4\,nJ}{1/1300\,Hz} = 0.590\,\mu W, \tag{4}$$

realizing low power consumption when operating at kHz tuning frequencies.

4. Error analysis

For the MEMS actuation data and Alvarez focal tuning data presented in Figure 3 and Figure 4 in the main paper, we estimate the corresponding error based on the uncertainties of the analysis methods and the physical camera limitations.

4.1 Uncertainties in edge detection of MEMS platform actuation

The infrared camera captures the actuated displacement of the MEMS platform induced by various voltages. We perform edge detection of actuated features at multiple locations in each video frame to analyze local displacements. The algorithm automatically omits outliers and

ambiguous readout due to camera defects or resolution limitations. Given the valid local displacements $x_1, x_2, \ldots, x_N$ with a standard deviation σ_x, the overall device displacement is calculated as their mean

$$\bar{x} = \frac{\sum x_i}{N}. \tag{5}$$

We estimate the associated uncertainty by the standard deviation of the mean $\sigma_{\bar{x}}$[1], calculated as

$$\sigma_{\bar{x}} = \frac{\sigma_x}{\sqrt{N}}. \tag{6}$$

Another source of uncertainty can come from the resolution limit of the camera, which limits the location reading accuracy to the distance corresponding to half of a pixel. Therefore, in the actuated displacement data presented in the main paper, the error bars have been calculated as the corresponding standard deviation of the mean from the edge detection results or the distance of a half camera pixel, whichever is larger.

4.2 Uncertainties in focal tracking of Alvarez tuning

The infrared camera we have used to monitor the Alvarez metalens has intrinsic artifacts and defects such as uneven stripes and dead pixels, as shown in Figure S5(a). They are visible in raw device images captured by the camera, as shown in Figure S5(b). In focal tracking, we analyze the in-plane distribution of intensities to identify the bright clusters near the center as the potential focal spot. All the potential focal spots found from images taken along the optical axis are then compared to search for the brightest spot as the focus and the corresponding image plane as the focal plane at the given actuation voltage. Therefore, it is crucial to remove the abnormalities in the images prior to focal tracking. Since the camera artifacts and defects are mostly static, we use an algorithm to extract their locations and intensity deviation relative to the background to correct the corresponding abnormalities in the raw device images. As shown in Figure S5(c), although there is some faint stripe residue left, probably due to random intensity noise intervening with the correction process, the majority of the abnormalities have been removed, producing images ready for focal tracking.

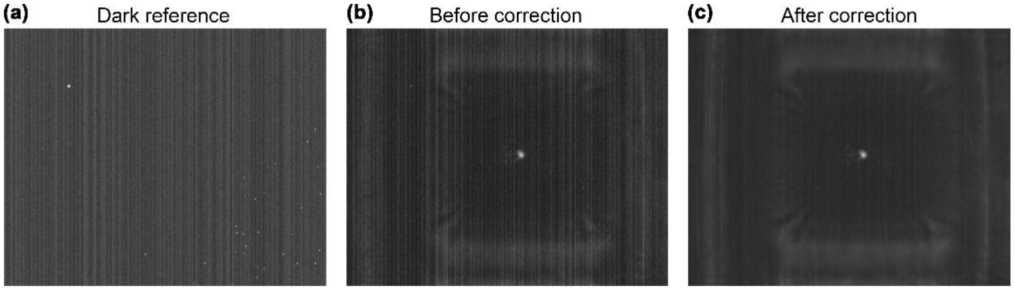

Figure S5. Image correction for camera artifacts. (a) Dark reference taken with no device in the optical pathway. (b) A raw example image taken at the focal plane. (c) The same example image with camera artifacts corrected using the dark reference.

However, just as the camera artifacts introduce local intensity offsets, the corresponding correction process will inevitably modify the captured pixel intensities at the potential focal spots, and the exact values of modification are affected by the artifact locations relative to the focal spots. Therefore, the most predominant uncertainty in focal tracking comes from the possible deviation in the search results caused by the spatially varying intensity abnormalities and the corresponding correction process. To estimate the range of uncertainties, we find the nominal focal plane first. Then we offset the intensity value of every pixel in the focal spot by a sigma of the dark reference image used to perform the image correction, mimicking the

extreme case of intensity deviation from the correction process. We search for the planes where the intensities of the potential focal spot decrease to the offset value, estimating the boundaries for uncertainties in focal plane tracking if the artifact removal process had locally over-corrected the local intensities around the focal spot.

References

1 Taylor, J. *Introduction to error analysis, the study of uncertainties in physical measurements*. 174-176 (1997).
